# Co-Design of a Neurodevelopment Assessment Scale: A Study Protocol

**DOI:** 10.3390/ijerph182312837

**Published:** 2021-12-06

**Authors:** Anne Masi, Syeda Ishra Azim, Christa Lam-Cassettari, Mark Dadds, Antonio Mendoza Diaz, Georgina Henry, Lisa Karlov, Ping-I Lin, Kylie-Ann Mallitt, Alicia Montgomery, Iva Strnadová, Andrew Whitehouse, Valsamma Eapen

**Affiliations:** 1 School of Psychiatry, Faculty of Medicine, The University of New South Wales (UNSW Sydney), Sydney, NSW 2052, Australia; a.masi@unsw.edu.au (A.M.); syeda_ishra.azim@unsw.edu.au (S.I.A.); c.lamcassettari@unsw.edu.au (C.L.-C.); a.mendozadiaz@unsw.edu.au (A.M.D.); lisa.karlov@health.nsw.gov.au (L.K.); daniel.lin@unsw.edu.au (P.-I.L.); a.k.montgomery@unsw.edu.au (A.M.); 2 Academic Unit of Child Psychiatry, South Western Sydney Local Health District and Ingham Institute, Liverpool, NSW 2170, Australia; 3 School of Psychology, Faculty of Science, The University of Sydney, Sydney, NSW 2006, Australia; mark.dadds@sydney.edu.au; 4 Cerebral Palsy Alliance Research Institute, Faculty of Medicine & Health, The University of Sydney, Sydney, NSW 2006, Australia; georgina.henry@sydney.edu.au; 5 Mental Health Research Unit, South Western Sydney Local Health District and Ingham Institute, Liverpool Hospital, Liverpool, NSW 2170, Australia; 6 Centre for Big Data Research in Health, The University of New South Wales (UNSW Sydney), Sydney, NSW 2052, Australia; k.mallitt@unsw.edu.au; 7 School of Women’s and Children’s Health, Faculty of Medicine, The University of New South Wales (UNSW Sydney), Sydney, NSW 2052, Australia; 8 School of Education, Faculty of Arts, Design and Architecture, The University of New South Wales (UNSW Sydney), Sydney, NSW 2052, Australia; i.strnadova@unsw.edu.au; 9 Disability Innovation Institute at UNSW, The University of New South Wales (UNSW Sydney), Sydney, NSW 2052, Australia; 10 Telethon Kids Institute, University of Western Australia, Nedlands, WA 6009, Australia; Andrew.Whitehouse@telethonkids.org.au

**Keywords:** neurodevelopmental disorders, neurodevelopment assessment, transdiagnostic, clinical phenotype, scale development

## Abstract

Neurodevelopmental disorders are a heterogeneous group of conditions with overlapping symptomatology and fluctuating developmental trajectories that transcend current diagnostic categorisation. There is a need for validated screening instruments which dimensionally assess symptomatology from a holistic, transdiagnostic perspective. The primary aim is to co-design a Neurodevelopment Assessment Scale (NAS), a user-friendly transdiagnostic assessment inventory that systematically screens for all signs and symptoms commonly encountered in neurodevelopmental disorders. Our first objective is to undertake development of this tool, utilising co-design principles in partnership with stakeholders, including both those with lived experience of neurodevelopmental disorders and service providers. Our second objective is to evaluate the face validity, as well as the perceived utility, user-friendliness, suitability, and acceptability (i.e., ‘social validity’), of the NAS from the perspective of parents/caregivers and adults with neurodevelopmental disorders, clinicians, and service providers. Our third objective is to ascertain the psychometric properties of the NAS, including content validity and convergent validity. The NAS will provide an efficient transdiagnostic tool for evaluating all relevant signs, symptoms, and the dimensional constructs that underpin neurodevelopmental presentations. It is anticipated that this will maximise outcomes by enabling the delivery of personalised care tailored to an individual’s unique profile in a holistic and efficient manner.

## 1. Introduction

Neurodevelopmental disabilities are a heterogeneous group of conditions including intellectual disability, motor, communication and specific learning disorders, tics, and conditions such as the autism spectrum, attention deficit hyperactive disorder, and cerebral palsy. Developmental and mental health disorders affect around 8% of children globally [1]. These children may experience a range of difficulties with attention, behaviour, language, learning, motor skills, social relationships, and other neuropsychological functions [2]. Comorbidities, or the co-occurrence of two or more different disorders, is common in children with a neurodevelopmental disorder [2]. Assessment and diagnosis of neurodevelopmental disorders often occur in “silos” for each domain and symptom in isolation, thereby needing the use of multiple questionnaires, interview schedules, and diagnostic criteria for each primary and comorbid condition. This approach is prone to the omission of important aspects of clinical characterisation or profiles (phenotypes) and may represent a duplication of efforts. Furthermore, it does not facilitate optimal understanding of complex presentations where more than one diagnostic condition co-exists. Rather than employing a transdiagnostic approach to neurodevelopmental assessment and management, the siloed approach encourages a culture of compartmentalising diagnoses to the point that they are considered mutually exclusive. Identifying appropriate and timely therapy and interventions for ‘downstream’ symptomatology is impeded and complicated by the current ‘ad hoc’ and ‘one size fits all’ approach to assessment.

Neurodevelopmental disorders have been historically considered as distinct entities and clinical conditions, each with a separate natural history and course of its own. The term Early Symptomatic Syndromes Eliciting Neurodevelopmental Clinical Examinations (ESSENCE) has been proposed to reflect the clinical reality of children presenting with a complex set of features that intersect a number of diagnostic conditions in childhood which share a trajectory of overlapping developmental or behavioural issues later in life [3,4]. There is a growing recognition that neurodevelopmental disorders share symptomatology [5], follow a fluctuating developmental course [3], and can vary over time; crossing, overlapping, or transcending current diagnostic categories and boundaries [6,7]. Further, neurodevelopmental disorders often share aetiological (genetic and environmental) and predisposing risk factors [8,9]. Thus, it would appear that these conditions are dimensionally distributed in the population [10] or, more precisely, that the categorical conditions exist along a continuum of neurodevelopmental differences [11]. These findings support an approach utilising validated screening instruments which dimensionally assess symptomatology from a holistic, transdiagnostic perspective rather than a battery of various compartmentalised assessments. Additionally, current diagnostic systems are faced with challenges stemming from concerns about diagnostic robustness. For example, data from the national Child and Adolescent Twin Study in Sweden (CATSS) have shown that, while the high cut-off for autism has remained stable over the last 10 years, the prevalence of autism, as officially recorded in the National Patient Registry, has substantially increased [12]. There is also evidence that, during a period of stability for diagnostic criteria, there was a reduction in the behavioural severity of individuals diagnosed with an autistic disorder [13]. Further, diagnostic substitution has been identified in autism [14] and, in the USA between 1994 and 2003, a decrease in the prevalence of intellectual disabilities has occurred in conjunction with an increase in the prevalence of autism [15]. Changes in diagnostic criteria over the past three decades may play a role in the increased prevalence rate of autism, although its causes remain controversial. Using a dimensional symptom profile to guide diagnostic assessment may provide a value of being future-proof in longitudinal studies, unhindered by changes in diagnostic criteria or classification systems that may emerge in the future.

A recent population-based study incorporating data from the western Sweden cerebral palsy register identified that 75% of children with cerebral palsy had impairments associated with other neurodevelopmental disorders [16]. This study highlights the complexity of cerebral palsy presentations and other neurodevelopmental disorders, demonstrating the multitude of permutations that can exist under one neurodevelopmental disorder diagnostic label. The impairments identified included intellectual disabilities (53%), speech disorders (54%), epilepsy (41%), neuropsychiatric impairments (32%), ADHD (21%), vision (19%), autism (18%), and hearing (8%) [16]. Given that: (1) neurodevelopmental disorders co-exist, and symptoms frequently overlap; (2) many comorbid neurodevelopmental disorders share common aetiology; and (3) associated comorbidities and mental health difficulties often cause the most distress and impairment, a transdiagnostic approach to the assessment of neurodevelopmental differences is critical. In this regard, it is noteworthy that phenotypic variability may be the result of neurodevelopmental genes converging on a common pathogenetic process, resulting in abnormal development that cuts across broad domains and disorders and yet having distinct cognitive and behavioural profiles [11].

Aim of this study:

The primary aim of this project is to co-design a Neurodevelopment Assessment Scale (NAS), a user-friendly transdiagnostic assessment inventory that systematically screens for signs and symptoms commonly encountered in neurodevelopmental disorders and flags any comorbid features so as not to exclude comorbid conditions in the screening process. By streamlining the early detection of neurodevelopmental differences as they emerge, this tool may assist clinicians in the timely identification of children who would benefit from early intervention, whilst also reducing clinician and parent/carer burden. It is expected that the innovative scale will allow a comprehensive assessment that will help flag the features relevant to the primary and any comorbid conditions and serve as a proxy for the presence of such conditions as this will allow further condition-specific testing and appropriate matching of intervention and supports.

Inclusion of consumer voices through co-design and co-production [17], with participants with lived experience engaged in all phases of qualitative stakeholder consultations (pre-design consultation, mid-design consultation, and post-design evaluation), and quantitative data analysis (ascertainment of the psychometric properties of the NAS using clustering of behaviours and symptoms, a validation sample, and post validation evaluation and adaptation) will be used to create the NAS.

The key objectives of this study are:

Aim 1: Co-design the development of a neurodevelopmental assessment scale (NAS) with stakeholders, including parents/caregivers of children with a neurodevelopmental disorder, adults with a neurodevelopmental disorder, health professionals and clinicians experienced in working with children with a neurodevelopmental disorder, and disability service providers, to facilitate a transdiagnostic approach to the comprehensive assessment of neurodevelopmental disorders, including any comorbid conditions.

Aim 2: Evaluate the face validity, as well as the perceived utility, user-friendliness, suitability, and acceptability (i.e., ‘social validity’), of the NAS from the perspective of clinicians, parent/caregivers, and adults with neurodevelopmental disorders.

Aim 3: Ascertain the psychometric properties of the NAS, namely, content validity and convergent validity.

## 2. Materials and Methods

### 2.1. Study Design Overview

This study will be conducted in three phases (Table 1). Each phase will contain a round of qualitative consultations and quantitative data analysis. Qualitative consultations with stakeholders or community representatives will be conducted using either online focus groups, interviews, or a semi-structured questionnaire to ensure the NAS is informed by lived experience at each stage of development.

### 2.2. Study Setting, Population, and Duration

Eligibility criteria for participants taking part in the stakeholder/community consultation component of the study include being (1) 18 years of age or older; (2) a parent/caregiver of a child with a neurodevelopmental disorder; (3) an adult with a neurodevelopmental disorder; or (4) a health professional or clinician working with children with a neurodevelopmental disorder or a representative of a disability service provider. Eligibility criteria for participants taking part in the NAS validation component of the study include (1) parents/caregivers of a child with a neurodevelopmental disorder who is preschool aged (ages 2–6), attending primary (ages 5–13), or secondary school (ages 13–18); (2) who are able to communicate in English; and (3) have reliable internet access.

Initial contact with potential participants for the stakeholder/community consultation component (Phases 1a, 2a, and 3a) and NAS validation component (Phase 2b) will be made via recruitment invitations circulated on social media and across the services and groups supported by disability service providers and advocacy groups. Recruitment invitations will also be sent to health professionals and clinicians and disability service providers and advocacy organisations using their publicly available contact details.

Potential participants can indicate their interest in participating by contacting the research team directly via email/phone using the contact details provided on the recruitment invitation and advertisement. Once a potential participant is confirmed to be eligible to participate in the study, the researcher(s) will undertake the consent process and arrange for the data collection process by advising the participant of the dates and times for the online focus groups, the options for an interview via phone or online, and also the option to complete the consultation as a semi-structured online questionnaire.

### 2.3. Study Procedures

#### 2.3.1. Stakeholder/Community Consultations Phase 1a

The first stakeholder consultation will be conducted to identify what should be included in the NAS and the barriers and enablers experienced by key stakeholders during the current assessment process. There will be 6–8 participants in each group of stakeholder consultations, an adequate number to reach data saturation as the stakeholders are knowledgeable, have lived experience, or are experts in the topic, and it is for a non-commercial study [20]. Participants in either the focus group or interview will be emailed questions one week before the scheduled consultation. Stakeholders choosing to participate via online questionnaire, administered via REDCap [21], will be emailed a link to the questionnaire. Easy-read versions of the questions will also be available. The provision of the 3 modes of participation (e.g., online focus group, online interview, online questionnaire) is aimed at providing options to participants so they can choose the format that is best suited to them and accessible for them.

#### 2.3.2. NAS Development Phase 1b

Deidentified data from data repositories including the Australian autism biobank [22], the autism cooperative research centre subtyping project [23], and the Child Behaviour Research Clinic [24] will be accessed. The phenotype data, which represent the symptomatology of children with a neurodevelopmental disorder who participated in the studies associated with these datasets, will be collated and consolidated for analysis. Relevant items will be extracted and divided into clinical and/or theoretically driven subscales. Clinically relevant subscales will be based on the symptomatology of presentations associated with neurodevelopmental disorders and will include dimensions such as: language; communication; perception; concentration and attention; impulsiveness and activity; learning; memory; planning and organising; social interaction; flexibility; compulsions; sensory processing and self-regulation; tics; sleep; pain; bladder and bowel control, separations; externalising symptoms; and internalising symptoms. Clinical medical characteristics which are not covered in the datasets will be included through the stakeholder consultation with clinicians and, specifically, will include items to cover motor type, tone, and function; motor and posture control; mobility, co-ordination, and gait; reflexes; involuntary movements, and feeding and swallowing difficulties; other bodily functions such as vision, hearing, and being severely overweight. Machine learning techniques and unsupervised clustering will be done to yield core items and subscales which will be assembled into an initial draft of the NAS.

#### 2.3.3. Stakeholder/Community Consultations Phase 2a

In this second phase, participants will be presented with the clinically and theoretically driven items and subscales developed during the analysis of existing datasets. The participants will be guided through a series of questions to facilitate an understanding of whether the items that comprise the NAS are deemed appropriate and relevant to each participant group. This phase will help participants to develop and clarify their perspectives about the utility of the clinical, theoretical, and statistically driven factors or constructs. In addition to seeking feedback on the relevance of items, the best way to capture the impact of item content will also be ascertained. For example, key questions addressing functional impairments may be relevant due to: (1) causing distress; (2) interfering with daily functioning; or (3) frequency and intensity. This will help assign a functional impairment level as mild, moderate, or severe. Wellbeing questions covering physical, psychological, and social wellbeing will be included to ascertain the quality of life, as well as a question framed to ascertain information around the child’s strengths.

#### 2.3.4. NAS Development Phase 2b

The purpose of this phase is to validate the NAS in preschool (ages 2–6) and school-aged children who have a neurodevelopmental disorder. The psychometric properties of the NAS in terms of internal consistency, test–retest reliability, and concurrent validity against another existing assessment used in children with a neurodevelopmental disorder will be evaluated. Future developments or refinements of the scale for general population and clinical use will also be identified. In addition to items addressing key areas of neurodevelopmental concern, the NAS will also include basic demographic and clinical questions such as age, gender, school grade/centre/other placements, and minimal information on the person completing the NAS.

Data will be collected through parent/caregiver completion of both the NAS questionnaire and an evaluation questionnaire. The evaluation questionnaire will be used to assess whether the parent/caregiver perceives each item should be included in the NAS and their opinion about areas for improvement of the questionnaire. Both questionnaires will be administered via REDCap, a secure, online survey platform. The concurrent validity will be ascertained by administering a semi-structured interview to parents/caregivers, the diagnostic interview schedule for children, adolescents, and parents (DISCAP; [25]). The DISCAP is a clinical diagnostic interview which is based on DSM classification. The validation will be conducted online (NAS and evaluation questionnaire) and via phone (DISCAP). A trained researcher will administer the DISCAP to complete the concurrent validity procedure. The DISCAP is a semi-structured interview based on the diagnostic categories of the DSM-5 affective/mood and disruptive disorders applicable to children and adolescents.

The NAS and parent/caregiver evaluation questionnaire will be completed by parents/caregivers of 100 children across 3 age groups that cover the range 2 to 18 years: preschool aged, primary school aged and secondary school aged (approximately 30 per age group). It is anticipated the NAS will take approximately 10–15 min to complete and the evaluation questionnaire will take less than 5 min to complete. Parents/caregivers will be asked for feedback on the wording of the NAS measure in the evaluation questionnaire, including identifying any items they do not understand and suggesting alternative wording for these, ensuring questions are clear, meaningful, and coherent. The evaluation questionnaire will include brief measures of parent demographics (e.g., age, sex, education, socio-economic status).

#### 2.3.5. Stakeholder/Community Consultations Phase 3a

**Figure 1:**
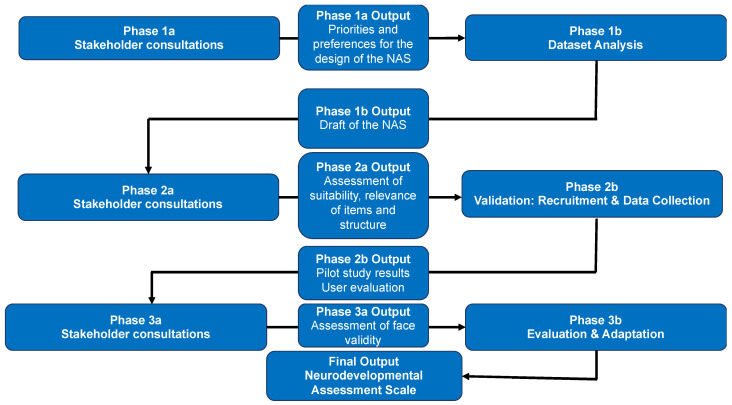
The flow of research activities through each of the three phases of the research process.

In this third phase, participants will be presented with the NAS that was administered to participants in the recruitment and data collection (Phase 2b—Table 1 and Figure 1). The participants will be guided through a series of questions to collect input on the face validity and user-friendliness (i.e., social validity) of the NAS, as well as health literacy screening to contextualise readability and comprehensibility.

#### 2.3.6. NAS Development Phase 3b

The data collected from the administration of the NAS during Phase 2b—recruitment and data collection in a validation sample—will be analysed in conjunction with the information collected from the stakeholder consultations conducted during Phase 3a. The NAS will be evaluated and adapted to produce a final version of the scale. No additional data will be collected in Phase 3b.

## 3. Analysis Plan

### 3.1. Phase 1a: Stakeholder/Community Consultations

The aim of the stakeholder consultations is to elucidate key themes around (i) the key diagnostic elements of neurodevelopmental disorders; and (ii) the face and social validity and suitability of the draft NAS. Interpretation and thematic analysis of the data collected during stakeholder consultations will be guided by the grounded theory method [26]. Identified themes will be compiled into a coding frame and, as new themes emerge, they will be compared against the initial coding frame and either added as new themes or used to expand and modify existing themes until all data are accounted for. Data analysis will be undertaken using constant comparison methods and matrix displays will be used to explore similarities and differences across groups on key themes [27]. The initial focus group and in-depth interview transcripts will be coded independently by two members of the research team to check the reliability of the coding frame. The data will be analysed using NVivo software for emerging themes [28].

### 3.2. Phase 1b: Analysis of Existing Datasets

The aim of analysing existing datasets is to identify the key components of neurodevelopmental disorders’ symptoms and move from groups of symptoms to a series of questions that will comprise the NAS using a logical and parsimonious framework, thereby offering an opportunity for targeted early intervention based on these common and unique symptoms present across major childhood disorders [29]. Statistical analysis of existing datasets will be conducted to ascertain clustering of behaviours and symptoms using ‘unsupervised’ machine learning techniques. An empirical, statistically significant latent class structure will also be pursued to test which of these different models, either in isolation or in a cumulative manner, will best predict homogeneous groups that are clinically meaningful and will allow matching interventions. Each subscale will be assessed across a wide range of different developmental diagnoses but without taking diagnostic exclusion or hierarchies into account. Clusters of symptoms will be determined using a model-based Bayesian information criterion (BIC) clustering algorithm based on expectation-maximisation (EM) algorithm for finite normal mixture modelling with different covariance structures and different numbers of mixture components.

### 3.3. Phase 2a: Stakeholder/Community Consultations

Similar to 1a, the stakeholder consultation data analysis will be based on the grounded theory method [26]. The consultation will be about the clusters identified in Phase 1b to identify clinically meaningful items for NAS along with subscales. Identified themes from the discussion about the clusters identified will be compiled into a coding frame and, as new themes emerge, they will be compared against the initial coding frame and either added as new themes or used to expand and modify existing themes until all data are accounted for. Data analysis will be undertaken using constant comparison methods and matrix displays will be used to explore similarities and differences across groups on key themes [27]. The initial focus group and in-depth interview transcripts will be coded independently by two members of the research team to check the reliability of the coding frame. The data will be analysed using NVivo software for emerging themes [28].

### 3.4. Phase 2b: Validation: Recruitment and Data Collection

Construct groupings using cluster analysis will be superimposed on diagnostic data using the best-estimate specialist diagnoses method for convergent validity. Receiver operating characteristics (ROC) will be used to illustrate the area under the curve (AUC) for the different scale steps. The total and subscale scores from the inventory will be used as independent predictors and the clinical diagnoses, as per DSM-5, will be used as dependent variables. For example, the dimensional construct of ‘impulsivity’, ‘arousal’, and ‘attention’ may best converge onto clinical ADHD diagnosis, in which case they will be grouped together to flag a diagnosis of ADHD. The cut-off value for each construct will be examined by measures of sensitivity, specificity, positive predictive value (PPV), and negative predictive value (NPV).

### 3.5. Phase 3a: Stakeholder/Community Consultations

Face validity and social validity will be obtained from the stakeholders through online focus groups or interviews or a semi-structured online questionnaire. The discussion will be compiled into a coding frame and, as new themes emerge, they will be compared against the initial coding frame and either added as new themes or used to expand and modify existing themes until all data are accounted for. Data analysis will be undertaken using constant comparison methods and matrix displays will be used to explore similarities and differences across groups on key themes [26]. The initial focus group and in-depth interview transcripts will be coded independently by two members of the research team to check the reliability of the coding frame. The data will be analysed using NVivo software for emerging themes [28].

### 3.6. Phase 3b: Evaluation and Adaptation

The aim of the evaluation phase is to ascertain the dimensional constructs that characterise the unique profile of an individual using NAS in a cohort of 100 participants with neurodevelopmental disorders. The participants will be of homogenously distributed age groups preschool (ages 2–6), primary school (ages 5–13), and secondary school (ages 13–18). The results from the NAS assessment will be superimposed on the clinician assessment of diagnostic categories to understand the overlap. This will be achieved using the diagnostic odds ratio, sensitivity, specificity, and Bland–Altman plots. We will assess demographic and clinical risk factors for congruence between the NAS and prior diagnosis using multivariable logistic regression. Convergent validity for the children assessed using the NAS will be ascertained against clinical diagnosis as per the DISCAP (which is based on DSM-5 Classification).

Analyses will be conducted using SPSS statistical software and Cronbach’s alpha values will be computed as per methods described in our earlier work with similar sample size [30]. Pearson’s product-moment correlations will be computed to examine the concurrent validity of NAS against clinician evaluation using standard clinical criteria. Alpha will be set at 0.05 for all comparisons, following recommendations by Saville [31] who argues for this per-comparison level rather than a family-wise approach when conducting research in novel areas. Stakeholder consensus on usefulness will be incorporated.

## 4. Discussion

A multidisciplinary and dimensional approach to comprehensive neurodevelopmental assessment, diagnosis, co-occurring symptoms, and difficulties that cause impairment in adaptive functioning, needs to be integrated with other associated aspects of an individual’s presentation, for example, genetic make-up and constitutional characteristics [32]. Furthermore, it is worth acknowledging that diagnostic overlap obfuscates other comorbidities, such as mental health or behavioural concerns, which are oftentimes reported as being responsible for more distress and impairment than the original diagnosis. In this sense, a transdiagnostic approach based on specific skills and behaviours may (1) better reflect and support the lived experience of the child and also the carers and families and (2) expedite the neurodevelopmental assessment process. It will result in consolidation of a number of validated assessment questionnaires and measures that are currently used in siloed approaches by different service providers, disciplines, and government agencies, thereby facilitating a more holistic mindset to the identification of each child’s overall but unique profile.

The Standardized Infant NeuroDevelopmental Assessment (SINDA) [33] is a recently developed assessment for infants aged 6 weeks to 12 months. The neurological scale includes five domains assessing spontaneous movements, cranial nerve function, motor reactions, muscle tone, and reflexes. Unlike SINDA, which focusses on motor function in the first year of life, the Neurodevelopment Assessment Scale will screen for a comprehensive range of signs and symptoms across the developmental years from 2 to 18 and cover all the domains commonly encountered in neurodevelopmental disorders, such as: communication, social interaction, flexibility, compulsions, symptoms related to sensory processing and self-regulation, sleep, and externalising and internalising symptoms. Furthermore, NAS will incorporate the child’s strengths and needs, offering a novel way to interpret the heterogeneity associated with various neurodevelopmental disorders.

## 5. Conclusions

Currently, there are no tools available for the comprehensive assessment of neurodevelopmental disorders and associated comorbid conditions. Alternatively, clinicians use specific rating scales for the main symptom presentation of individual neurodevelopmental disorders such as for ADHD, anxiety, obsessive compulsive disorder, autism spectrum, and tics. The NAS will replace these different scales and measures by providing a single transdiagnostic tool, facilitating a transdiagnostic approach to elucidating and evaluating all neurodevelopmental signs, symptoms, and the dimensional constructs that underpin them in an efficient manner. It is expected that this will maximise outcomes by enabling the delivery of personalised care, so each child receives intervention and support services in individualised ways based on their unique profile at the earliest opportunity to derive the most benefit from intervention.

## Figures and Tables

**Table 1:** Description of the three phases and two components of the study.

	Stakeholder/Community Consultations	NAS Development
Phase 1	Initial consultation conducted separately with the following groups: parents/caregivers of children with a neurodevelopmental disorder; adults with a neurodevelopmental disorder; health professionals and clinicians experienced in working with children with a neurodevelopmental disorder and disability service providers.	Cluster analysis of existing databases containing diagnostic and functional assessments of NDDs analysed to ascertain clinical and theoretical subscales on the NAS. Items will have a readability level of grade 8 or lower [18].
Phase 2	Consultation with stakeholders from Phase 1 to review outcomes from dataset analyses (Phase 1b) to identify clinically meaningful items for NAS along with subscales. Parents/caregivers will be asked whether they feel each item should be included in the NAS and to provide their opinion about areas for improvement in the NAS.	Validation: recruitment and data collection in a pilot study of the NAS with a sample of parents/carers of children with NDDs who are preschool aged (ages 2–6), or are attending primary (ages 5–13), or secondary school (ages 13–18).
Phase 3	Consultation with stakeholders from Phases 1 and 2 to assess face validity and user experience/friendliness (i.e., ‘social validity’). Social validity is defined as a measure of the overall acceptability of a measure, tool, or intervention beyond its effectiveness as perceived by the people who are implementing, receiving, and consenting to it [19].	Data analysis: NAS validation. Evaluation and adaptation to finalise the NAS.
